# Enhancing patient-provider relationships with a whole person oriented healing pathway model

**DOI:** 10.1186/s12913-025-12858-8

**Published:** 2025-05-13

**Authors:** Jolie N. Haun, Justin T. McDaniel, Rachel C. Benzinger, Christine Melillo

**Affiliations:** 1 Research and Development Service, James A. Haley Veterans’ Hospital, Tampa, FL USA; 2 Division of Epidemiology, Department of Internal Medicine, University of Utah, Salt Lake City, UT USA; 3 School of Human Sciences, Southern Illinois University, 475 Clocktower Drive, Carbondale, IL 62901 USA; 4 Seattle-Denver Center of Innovation, Rocky Mountain Regional VA Medical Center, Aurora, CO USA

**Keywords:** Caring science, Healing, Humanistic, Integrative health, Model, Relationships

## Abstract

**Objective:**

This paper identifies provider characteristics, across medical specialties, that facilitate a healing pathway model for patients.

**Design:**

With a phenomenological approach, a prospective descriptive study design was used to conduct in-depth semi-structured focus groups and individual interviews, which elicited experiences facilitating healing. Thematic content analysis methods were used to organize and analyze data findings within the context of a healing pathway model.

**Setting:**

Data were collected in three geographically diverse areas representing various fields of practice in conventional and complementary and integrative health (CIH).

**Patients or other participants:**

Snowball sampling was used to collect data from 52 providers from diverse healthcare settings.

**Results:**

As a group, participants described three healing domains, including (1) Provider Approach for Facilitating Healing; (2) Foundations of a Healing Pathway; and (3) Observation of Healing Outcomes.

**Conclusions:**

As the dynamics of healthcare continue to become more complex, and consumeristic in nature, constructs emerge across disciplines reflecting an interpersonal approach to facilitate healing. These emergent constructs informed the development of a conceptually driven healing pathway model to identify points of intervening and informing how to leverage patient-provider relationships to facilitate healing.

**Supplementary Information:**

The online version contains supplementary material available at 10.1186/s12913-025-12858-8.

## Introduction

The whole person health approach to integrative medicine is becoming commonplace in United States healthcare systems [1, 2]. However, as Jonas and Rosenbaum noted, “definitions are quite heterogenous and … there is a need for more standardization of whole-person models and more research using whole systems approaches rather than reductionistic attempts using isolated components [2]. Whole person health suggests health and disease are not mutually exclusive but rather a pathway, characterized by reciprocity and feedback, and influenced by multiple levels, such as biological, behavioral, social, and environmental factors [2, 3]. As the nation’s healthcare systems undergo dramatic shifts toward a patient-centric paradigm, the patient-provider relationship is a point of intervention for optimizing health outcomes [4–8]. With a whole health approach to care, providers can connect with patients using a humanistic approach to optimize potential for supporting wellness [9, 10].

Whole health oriented, patient-centered healthcare emphasizes interpersonal dynamics, mutual participation in decision-making, use of appropriate resources, reciprocity and feedback, and patient education [11]. Patient-centered care can be best understood using a systems paradigm, such that healing is catalyzed through an active process of information transformation, feedback, and ongoing ever-evolving relations between patients and their providers. The representation of a complex system, such as the one previously described, is best represented via a pathway model [12–16].

Pathway models in healthcare can depict the entire course of a patient’s treatment, from initial diagnosis to recovery [13, 14]. For example, in managing chronic diseases, a pathway model might include stages such as initial consultation, diagnostic tests, treatment planning, therapy sessions, follow-up appointments, and ongoing monitoring [15, 17, 18]. Each stage can be broken down into responsibilities, processes, and expected outcomes. A pathway model facilitates communication among multidisciplinary teams, ensuring that every professional involved understands their role in the patient’s healing pathway. When introduced to the patient, pathway models can enhance patient engagement by providing them with a visual representation of their treatment plan.

Beyond recognizing the patient-provider interaction as a complex system which is continuously evolving, the interaction should be illustrated to capture aspects of exchange between patients and providers from a provider perspective [19, 20]. As such, the present paper draws upon pathway modeling strategies to represent the data explored in this project. The purpose of this paper is to identify self-perceived provider characteristics which facilitate the whole person healing pathway across health professions and to guide development of a conceptual pathway model for evaluating the patient-provider relationship as a point of therapeutic intervention. The primary research question addressed in this study was: How do providers perceive that they are facilitating the whole person healing pathway and promoting continuous healing relationships in their practice?

## Background

The patient-provider relationship has significant impact on patient outcomes, including participation, decision-making, adherence, litigation, resource use, quality of life, satisfaction, and clinical outcomes [21–28]. Though theoretical pathway models and measurement instruments commonly address aspects of the patient-provider relationship including satisfaction, trust, well-being and wholeness, understanding, confidence, and global satisfaction [24, 29], the literature is deficient on critical aspects of the continuous healing relationship between patients and providers. Current descriptive models and instruments used to assess the patient-provider relationship are limited in two distinct ways. First, they are typically limited to nursing and physician providers, rather than a diversity of conventional and complementary and integrative health (CIH) providers. This is important because CIH providers play a significant role in current integrative health care models [30]. Second, current pathway models are limited in the constructs they address – that is, they only focus on communication style, engagement, and decision-making [31–34]. This paper presents data and a preliminary pathway model to organize emergent concepts that represent how providers perceive they facilitate healing.

### Patient-provider relationship: theoretical context

Understanding relevant factors of the patient-provider relationship informs timely, efficient, safe, equitable, and effective patient-centered practice [35]. Healing relationships, effective communication, and shared decision-making between patients and providers are identified as key interpersonal components of patient-centered care [36, 37]. We propose to use the Mutual Participation Model [38], which defines the provider’s role as the patient’s expert helper. The provider helps a patient help themselves. The patient’s role is as an active partner that participates in the decision-making process. The advantage of the Mutual Participation Model is that the patient and provider are in mutually supportive roles with shared responsibility for the healing pathway, and they are cooperative in achieving the patient’s health goal(s) [19, 20].

Previous models [38–41] have been published to guide precision and value for shared decision-making and communication between patients and providers, focusing on diagnosis, treatment option exploration, treatment decisions, implementation, and evaluation; however, said models do not build on relationship development [40]. The Rede Model [39] emphasizes the relationship development process relying on traditional aspects of building rapport, engagement, elicitation, and collaboration; however, it was Watson’s work that advanced these models toward a more caring science approach to relationship building and nurturance over time. Watson’s extensive work serves as a framework for the support of a “caring occasion,” [42] which is based on five core principles [43]: (1) practice of loving-kindness and equanimity; (2) authentic presence: enabling deep belief of other (patient, colleague, family, etc.); (3) cultivation of one’s own spiritual practice toward wholeness of mind/body/spirit; (4) “being” the caring-healing environment; and (5) allowing miracles (openness to the unexpected and inexplicable life events).

These published theoretical models and frameworks for advancing caring science provide a critical theoretical context for understanding continuous healing relationships from the patient and provider perspective. The current study builds on these previously developed theoretical models (e.g., Watson’s model, the Rede model). Yet, based on current gaps in the literature, it is important to further examine how diverse healthcare professionals – from conventional and CIH practices – perceive their role in facilitating healing along a pathway. These data can inform the development of whole person health models of care, from the provider perspective. The development of a literature and data informed Health Pathway Model expands the scope and nature of models used to characterize the patient/provider relationship. We propose abstract theoretical concepts that go beyond the traditional relationship and communication models in health outcome research. Our model could be used in health communication research, patient education programs, and anthropological research efforts aimed at continuing the advancement of the science of understanding the human impact on health care.

## Methods

### Study design

We employed a phenomenological approach to understanding the lived experience of participant providers in facilitating healing [44–46]. We chose a prospective descriptive study design to conduct focus groups and individual semi-structured interviews with conventional and CIH providers. Study procedures and all research activities were originally approved by the University of Arizona Institutional Review Board (IRB) and then re-approved at the lead authors academic affiliation, the University of South Florida Institutional Review Board.

### Sample and sampling

Health care professionals were recruited as participants using a snowball sampling technique in geographically diverse areas (Arizona, Florida, Georgia) in the United States. Professional affiliations with University of Florida, University of Arizona, Veterans Health Administration in the Southeast United States, and several CIH-focused teaching organizations were leveraged to make contacts with healthcare professional networks. Though snowball sampling was used to generate a participant sampling pool throughout these professional networks, purposive sampling was used to recruit participants representing diverse fields of practice in (1) conventional medicine; and (2) CIH. Contacts were made using phone calls and emails to share project details, acquire assent to participate, and schedule in-person contacts for scheduled consent and data collection. Inclusion criteria included being a conventional and/or CIH provider, of 18 years of age, with ability to speak English and to provide consent. Sampling of participants was conducted until data saturation was met within and across the two primary cohorts (i.e., conventional, CIH).

### Measurement and data collection

This study used in-depth, semi-structured individual and small focus group interviews to elicit health professionals’ experiences communicating and facilitating healing with patients. Participants were primarily offered the option for in-person individual interviews, however due to scheduling demands, some networks of participants requested to meet as a small, focused discussion. In these instances 2–5 participants were scheduled at their convenience for a small focus group discussion. Small focus groups included peer participants, without inclusion of superiors and/or leadership to avoid perceptions of power differential dynamics. In instances when participants held a leadership role, they were interviewed as individual respondents.

Individuals and small groups responded to items from a semi-structured interview script. Iterative design was used to develop the script items, representing theoretical perspectives of previous patient-provider relationship models with the integration of whole person oriented healing constructs [47]. The script was iteratively reviewed and revised with the guidance of research team members, who represented the targeted sample population, i.e., healthcare professionals from conventional and CIH medicine. The script was piloted with a collaborative research partner; minor revisions were made to finalize for data collection. Upon data collection launch, minor adjustments were made to enhance the script flow and resultant data acquisition. The final script contained 11 open-ended questions (Appendix A). Questions addressed relevant topics, including professional experience and years of practice; experiential aspects of working with patients; identifying patient needs; the pathway of facilitating care; perceived shifts in patients’ experiences; the pathway of co-creating healing; strategies in facilitating healing; and cleansing routines.

### Analysis

Thematic content data analysis methods were used to organize and analyze data [48]. Content analysis was used to identify patterns of similarities and differences by professional type. The analysis focused on descriptions of relationships and recurring patterns of experience, behavior, and beliefs so as to identify domains and taxonomies related in provider interviews and focus groups [49]. Participant comments were organized to develop codes and then merged to develop categories. Categories were compared and contrasted using the constant comparative method, and relationships were identified across categories [50]. Categories were then grouped to create domains and taxonomies, data samples were extracted and coded by research team members and evaluated for inter-rater reliability and credibility. The conventional medicine and CIH provider data sets were compared to determine commonalities and differences. The research team conducted a complex matrix analysis to analyze across-group domains and taxonomies [51]. Descriptive and comparative matrices, which identified the patterns of regularities (shared) and inconsistencies, were then constructed by provider type [52]. This descriptive and comparative analysis allowed discernment of salient and representative data. Exemplar and representative cases were extracted from the dataset and analyzed for domains and themes. Following qualitative data analysis, we visualized the relationships between thematic constructs and values, as well as contextual factors, in a pathway model, based on a contextualized literature-informed approach [50, 53].

## Results

Data were collected with provider participants (*n* = 52) in eight focus groups (*n* = 25; 2–5 participants/focus group), and individual interviews (*n* = 27). There were 12 male and 40 female participants. Participants reported an average of 20.4 years of professional practice experience (Range = 1–40); and identified as CIH providers (e.g. massage therapists; acupuncturists; naturopathic physicians), conventional medicine providers (e.g. nurses; physicians; nutritionists), or interdisciplinary CIH and conventional providers (e.g. nurse practitioner and Traditional Chinese Medicine). Notably, it was not common for conventional providers to practice multiple disciplines, as these providers are often highly specialized (i.e., OG/BYN, ear/nose/throat, radiology, dermatology, oncology, pediatrics). Participants reported the length of time (minutes) for patient visits; reports from CIH only (*n* = 19; M = 97, SD = 45) and conventional only (*n* = 23; M = 41, SD = 25) providers indicated that CIH providers spent more than twice as much time with patients than conventional providers. Provider type descriptions are provided in Table 1.

**Table 1:** Provider participants descriptive statistics

Provider Type	*n* (%)	Mean Years of Experience	Practice
Conventional Providers (single practice)	25 (48)	23	▪ Nursing ▪ Medicine (MD) ▪ Clinical Dietician
Conventional Providers (multiple practices)	0	0	
CIH Providers (Single practice)	13 (25)	16	▪ Massage Therapist ▪ Spiritual/Energy/Intuitive Healer ▪ Naturopathic Physician
CIH Providers (Multiple practices)	6 (12)	24	▪ Spiritual/Energy/Intuitive Healer (2) ▪ Massage Therapist/Feldenkrais^®^ ▪ Massage Therapist/ Kinesiology/Spiritual-Energy Healer ▪ Spiritual/Energy Healer/Hypno-therapist ▪ Traditional Chinese Medicine/ PSYCH- K^®^/Aromatherapy/Homeopathy/Neuro Emotional Technique™/Quantum Touch^®^
Mixed Conventional and CIH Providers	8 (15)	20	▪ Traditional Chinese Medicine/Nurse Practitioner ▪ Massage Therapist/Doula/Nurse/Exercise Physiologist ▪ Clinical Social Worker/Massage Therapist/Feldenkrais^®^ ▪ Massage Therapist/Personal Trainer ▪ Medicine (MD)/Massage Therapist ▪ Physician’s Assistant/Physiologist/Massage Therapist ▪ Nurse Practitioner/Arts in Medicine/Spiritual-Energy Healer ▪ Pharmacist/Massage Therapist/Spiritual-Energy Healer

Provider reports gleaned three primary domains related to facilitating healing across CIH, conventional, and interdisciplinary providers (i.e. CIH and conventional): (1) *Provider Approach for Facilitating Healing*; (2) *Treatment*; and (3) *Observation of Healing Outcomes*.

An overarching theme that is foundational to each of these domains is *Time as a Factor in Healing*. Participants mentioned time as a mediator of the healing pathway: time to deliver care, time to pause or participate in rituals before healing, allowing time to pass as one engages in a healing pathway, and time as a continuum within which they contextualize their practice. Though time potentially represents an obvious factor, it is worth mentioning that participants recognize the importance of time as a part of the healing pathway.

### Provider approach for facilitating healing themes

Provider approach descriptively reflects the way in which the providers reported similarities in how they generally facilitate healing. Themes within this domain include: (1) Facilitator; (2) Compassion and Presence; (3) Creating Healing Space; (4) Engaging the Whole Person; (5) Internalizing Shared Healing Experience; and (6) Self-Care.

These approaches describe how CIH, conventional, and interdisciplinary providers perceived their role as facilitators and partners in creating a healing pathway and their self-awareness of creating a compassionate presence. Respondents also reported the practice of creating a healing space both figuratively and literally. Additionally, they reported engaging the whole person, as opposed to only addressing symptoms, during the healing pathway. Providers also reported a common practice of cleansing themselves and committing to self-care to support their own wellness and maintain their capacity to facilitate healing. Exemplar excerpts – across specialty areas – representing the constructs are illustrated in Table 2.

**Table 2:** Provider approach for facilitating healing pathway themes

Domain	Sample Quotes
Provider as a Facilitator	I feel like I’m a teacher, a facilitator. I’m here to facilitate, to educate to help people have more productive lives. (Conventional)
	I view my role as a facilitator, meaning I have skills, I create an environment. I help that happen. (Interdisciplinary)
	As a facilitator. As one who helps, not one who does. (CIH)
Compassionate Presence	I try to remind myself to be compassionate, be a good listener, to try not to be judgmental as much as possible. To really be there and hear what the patient has to say. (Conventional)
	You know, people who are in the field of human touch…there’s a compassion and a love that develops that opens your own heart in service is the only way I can put it. You can’t help but feel for them. (CIH)
Creating Healing Space	It’s an environment that is calm, nurturing, and people walk in with that attitude. In a medical setting I fight the ‘get them in, get them out’ attitude. It’s the pressure put on us by the medical society. (Conventional)
	Because of the needs of the persona and the needs of the body, getting back to healing, I do consider myself a healer, small “h”. Not with the big ego, not like I touch you and you’re blind and now you see…But healer in the sense that I have cultivated myself to create a healing space where when somebody walks in their being immediately relaxes. And they feel safe, and they feel comfortable. (Interdisciplinary)
	I hold a space, which makes it possible for people to lift out of where they are into something better and that gives them a perspective and an ability to look at themselves. (CIH)
Engaging the Whole Person	We normally see our patients every four weeks to six weeks…we really get to know the patient; we get to know their lifestyle…what’s going on in their lives. (Conventional)
	…getting the knowledge medically and knowing the body physically, knowing the anatomy and educating myself enough to be able to speak it in lay terms and spiritual terms, has brought that bridge together and integrated every part of ourselves, emotional, physical, mental, spiritual and all levels and putting all that together makes a nice package to facilitate a nice session of healing. (Interdisciplinary)
Internalizing Shared Healing Experience	I feel like they are my sister or my brother, it’s such an intense connection, it’s a spiritual connection, is what it feels like, and it feels like such deep compassion. Compassion’s not even really the word for it. It’s like a soul connection. (Conventional)
	It’s going on a vision quest. I think the most important thing is giving the conventional health care provider the opportunity to explore their own inner life and their own life as a vision quest. On a much deeper level than just intellectual. I think it needs to be spiritual, I think it needs to be emotional. I think it needs to be physical. (Interdisciplinary)
	Oh sure. I can feel their emotions and sometimes you feel like what they’re going through. I can feel their feelings and then I feel my own. (CIH)
Self-Care	I go home and stay with my family and play with my kids. I do some gardening and some stuff and that releases the pressure. (Conventional)
	If something really negative happened maybe I’ll sponge a room out. But often, I am washing my hands or just taking a few breaths. (Interdisciplinary)
	At the end of a full day before I leave and lock up my office, I sit in my treatment space and I kind of just go over my day and let everything kind of go. (CIH)

### Treatment

Provider participants reported general practices during their interactions with patients during treatment, not specific to their scope of practice, to inform their process of facilitating healing. The four sub-domains within the treatment domain include: (1) Observation; (2) Building Rapport; (3) Resource Management; and (4) Communication.

Understanding how *observation* facilitates healing within the context of the patient-provider relationship is a critical component to the Healing Pathway Model. *General Assessment* is a construct conceptualizing the phenomenon that providers evaluate their patients while they navigate the pathway of healing. What may be more unique is the understanding of *Leveraging Intuition and Insight* and *Monitoring Energetic Changes* to understand the patient’s experience of healing. These are more metaphysical elements of observation and healing. These methods of observation can be utilized by all providers when fully present and engaged with the whole person. Once assessment is complete, *Identifying Needs* of the patient can occur, which includes not only their biomedical needs, but also their health and wellness goals.

*Building Rapport* is valued as a foundational attribute of the Healing Pathway Model as it supports mutual participation. A relationship of trust and *respect* is built upon clear communication. When patients and providers communicate, the provider is more likely to understand and respond to the patient’s needs and expectations, leading to patient *empowerment*. *Love* is also at the core of this interpersonal interaction. Although, to date, this concept has received little attention in clinical research, it is fundamental that love heals [54]. Love was seen as a tool for healing in this study. Providers addressed their patient’s need for love and sought to understand the patient’s quality of intimate/social relationships to identify emotional support needs for the patient. The provider’s ability to practice empathy through presence and acceptance was central to creating rapport and *connecting with the patient*. *Empathy* was an important factor for providers when reporting about interacting with patients. Empathy is valued as a foundation to increasing communication and patient participation. Empathy tended to decrease patients’ level of anger. Conversely, patients in un-empathic settings often demonstrated anger. When providers demonstrated empathetic behaviors, patients were more likely to disclose, feel secure, feel less anxious and be more confident in the availability of their practitioner. As such, connecting with the patient created a bond throughout the healing pathway. Connecting with patients was thought to be associated with psychological adjustment and engagement in treatment decision-making. Providers were able to connect with patients and maintain presence, acceptance, and empathy. They equipped themselves with the tools to optimally interact with patients on a personable level which promoted honesty and trust. *Empowerment* was also reported as central in promoting patient self-efficacy and managing expectations throughout the healing pathway. Patient empowerment was vital for individuals to facilitate the pathway toward achieving outcomes in their healthcare encounter [55]. As the patient experiences the previously mentioned rapport building mechanism, trust begins to develop between the patient and provider.

*Resource Management*, including providing *education and service referrals*, optimizes the patient’s preventative care and healing pathway. When providers were engaged and aware of individual patient needs, they collaborated with the patient to meet their information and resource desires. Service referrals, which promoted integration of healing systems, were the providers’ responsibility. These referrals provided patients with safe and effective options for additional treatment. Providers and patients worked as a team to gather information about the patient’s medical history and current health practices to integrate all pertinent information.

*Communication*, including *clear communication* and *assessing comprehension*, is readily recognized by participants as a primary factor in addressing health care quality. Interpersonal communication skills are necessary for both patients and providers, assuming that the communication process is an interchange where both parties influence the pathway and outcome. The ability to exchange and use information was reported as influential on health behaviors and outcomes. Implementing supportive practices (e.g., simple terms, alternative resources) decreases the stigma associated with needing assistance and emphasizes the importance of understanding health materials in a shame-free environment.

These sub-domains, their relevant themes and exemplar quotes, are illustrated in Table 3.

**Table 3:** Foundations of the whole person oriented healing pathway domains

Domain	Theme	Sample Quotes
Observation	General Assessment	I think that some of the patients that I meet are really easy to read. You can read them like a book. You can just open up the page, and it’s all there. But some of the others are really hard to read. (Conventional)
		I like to have them read themselves…I ask people to walk, and I try to have soft eyes and really soft intention. (Interdisciplinary)
		Talk to them. Ask them why they came? If you’re listening and you know what you are listening for then you hear it. (CIH)
	Identifying Needs	I do an age-old thing- I talk to them. An ancient secret- ask the person with the body what they want and what they’re experiencing. (Conventional)
		In massage, if they’ve got a multiple of multiples, we try to pick out one or two that are the most significant to them. In medicine, the same thing. What are their biggest concerns. (Interdisciplinary)
		Ask them before they go into a session. What’s your purpose? What’s your reason? What’s your needs? (CIH)
	Leveraging Intuition and Insight	(I knew) there’s something wrong with the baby. The APGARs [assessment of overall newborn wellbeing] were good, everything was good, but the baby just didn’t feel right to me. So…intuitively I knew it. Sometimes it would get me in trouble because I’d really force the issue that I thought something was wrong with the baby. (Conventional)
		I say through my prayers that I feel it becomes more awareness of the day and point of time I’m at in that moment. (Interdisciplinary)
		I normally don’t see anything until I’ve laid hands on them. That’s where I receive what’s happening in that person’s systems. (CIH)
	Monitoring Energetic Changes	When you begin to work with a client, you actually can touch the energy, you can move it. You can reach right in and move. (Interdisciplinary)
		I would say I’m a conduit, an electrical conduit. Where you open and ground, like an electrical conduit, and you open to light and grace, and it can feel like a vibration as well as a light. It can feel like a color vibration and when you open it’s a very humbling experience because you know how real it is. You know how real it is for the people so, it is a feeling of being truly of service. (CIH)
Communication	Clear Communication	There are times when I go over something with a client, and I feel it very strongly. We also have tears, where energy escapes, also. I will talk with that client, patient, after the session, and tell them what I found and where. (Conventional)
		I always talk very clearly and not use very many technical terms…if somebody says they don’t understand something, I feel like I’ve described it very simply and plainly, I just try a different route. (Interdisciplinary)
		Talk to them. Ask them why they came. If you’re listening and you know what you are listening for then you hear it. They don’t always know but if you listen then you pull out of what they’re saying what the core is. (CIH)
	Assessing Comprehension	Repeat facts…repeat back, then I explain it, and they were able to say yes, this is what this is. (Interdisciplinary)
		That age-old secret- I ask them. I talk to people. “Do you understand why that would be valuable for you to take that three-pound weight and do that. Do you understand, you know I just ask them? (CIH)
Building Rapport	Trust	It’s very hard to individualize patients because you’re always under a time frame. They want to get the patients in, get their reminders done, and get them ready for their providers. (Conventional)
		You have to learn to trust what you sense, see, feel, trust that goes beyond the dimensions of taste, sound, hearing. It’s trusting, trusting in something greater. Also trusting that we are all connected. We think we’re separate because we are in separate bodies but we’re really all connected. (CIH)
	Love	I feel like they are my sister or my brother, it’s such an intense connection, it’s a spiritual connection, is what it feels like, and it feels like such deep compassion. (Conventional)
		It’s a very deep, spiritual love in essence. (CIH)
	Empathy	It’s really hard to stay empathic…even though, I’ve had people in hospice die, I know it’s going to happen. But they haven’t had it happen to their mother. This is their mom. (Conventional) I can feel their emotions and sometimes you feel like what they’re going through. I can feel their feelings and then I feel my own. (CIH)
	Respect	…you work with the patient, you know you don’t just dictate to them all the time, you help them figure out what they need, you know, they help you figure out what they need. Then you work together to try to get it done. (Conventional)
		My goal with a client is to be 100% with them and to hear them. (CIH)
	Connecting with the Patient	You develop a bond. You develop a relationship, with these patients…and you will see them in the area, and other areas, and they will always have a kind word, and you know, want to make sure that you acknowledge them. (Conventional)
		I’m simply in relationship with my client. I’m having a dialogue with them, with their body. (CIH)
	Empowerment	You don’t try to interpret, you listen to what they say, because what they’re saying is their truth at that time, and you listen with your heart and connect with their energy and feel their energy where they’re coming from. (Conventional)
		…. the way a person describes themselves and their experience changes. Certainly, her experience of herself changed quite a bit. Her confidence changed. (CIH)
Resource Management	Education and Resources	They’re just more educated, and they know they can tell you about carbohydrate counting, when they couldn’t even tell you what a carbohydrate was before…a lot of its education. (Conventional)
		The new patients have to be educated about what to pay attention to, what the work is about…it takes a few lessons to figure out what’s going on with the FC work. (CIH)
	Service Referral	If a client…isn’t symptomatically improved to a level where they’re satisfied, then I refer them to other types of people who do the other things to get them up to the next level. (Interdisciplinary)
		…if they’re going to bring it up and I feel like they need to go see counseling at another level then I’ll refer them to counselors at another level, but the fact that it’s coming up and they’re hitting their own resistance within themselves, that’s usually an indication that there is something. I might just gently say, “Ok this is coming up for a reason so there is something for you to see here that this is happening” and not quell the moment. (CIH)

### Observation of healing outcomes

The third and final domain addressed providers’ shared perception regarding their observance of healing domain outcomes, which consist of: *acute shifts*,* healing*,* and things that prevent healing*. These observations about healing provide insight into how patients navigate the healing pathway. Exemplar quotes representing these constructs are illustrated in Table 4.

**Table 4:** Observation of healing outcomes domains

Domain	Sample Quotes
Acute shifts	I’ve had patients like be at death’s door one day, and the family come in and pray with them, or the, the priest come in and pray with them, and they would have like a turn around. And it, it was noticeable within twenty-four hours they would be a whole lot better. And then they would get better and go home. But it was like, kind of like night and day, kind of difference. (Conventional)
	But healer in the sense that I have cultivated myself to create a healing space where when somebody walks in their being immediately relaxes. And they feel safe and they feel comfortable. (Iterdisciplinary)
	I was wearing a hematite necklace, hematite is known to absorb negative energies. Well my necklace exploded, pieces hit the wall. And my hands on her liver and I thought, ‘Hmm, interesting.’ She opened her eyes and said, ‘What happened.’ And I said, ‘Well my necklace just broke.’ And she said, ‘No, no, no. The minute you touched me an electric charge went from your hand up my toes.’ (CIH)
Healing	It’s verbal, the patient voices it. Observation, lab values, tests, all those things which show improvement. (Conventional)
	I can get that adhesion to completely release, you know, often times there’s a POOF, you know, kind of thing. And they go, “Oh my God, that was it!” You know, it’s like, that was it. (Interdisciplinary)
Things that Prevent Healing	…preconceptions in terms of what to expect or problems in communication between the patients and the provider. And past experiences or bad experiences from the provider or the patient’s standpoint. Those are barriers that we encounter quite often in the healing process. Also, the severity of diseases and the history, the medical history and the social history of the patient as well as the provider. (Conventional)
	I’d have to toss it up to one of two things: Either I’m just, either my intuition or my intellect is just failing me. (Interdisciplinary)
	Sometimes people get in the way of their own healing. (CIH)

### Proposing a whole person health oriented healing pathway model

**Fig. 1:**
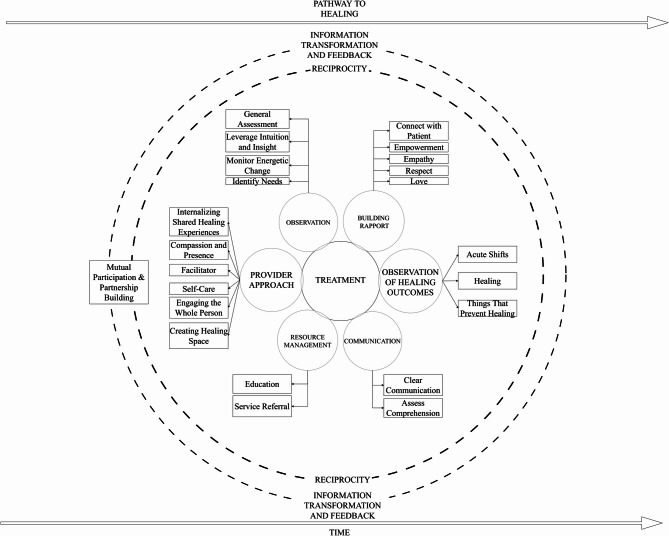
Data drive whole person health oriented healing pathway model

**Fig. 2:**
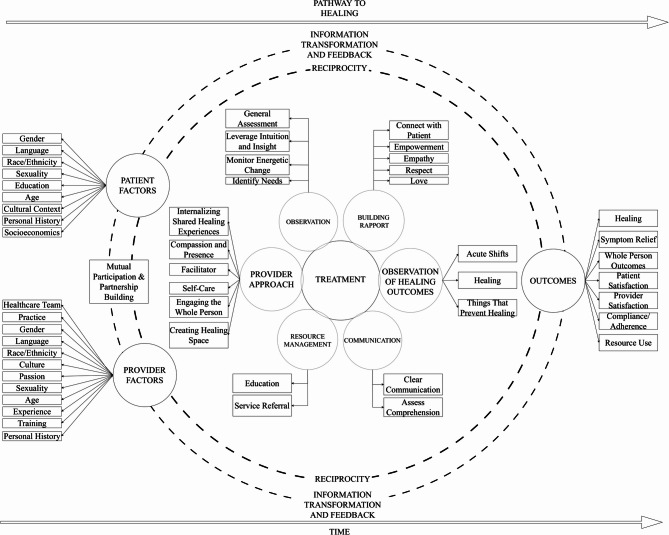
Data and literature-informed healing pathway model

Data-derived domains produced in this study provide a descriptive basis for the contextualization of the healing pathway within the patient-provider relationship. The following relevant constructs and data elements have been organized to represent a healing pathway through interpersonal factors. These factors have been illustrated in Fig. 1. Additionally, based on a literature-informed framework development approach, we refined the model to contextualize our empirical data within the broader literature on whole person health and healing (Fig. 2). It is clear from observing these two models that the literature supported components include patient and provider factors, as well as healing outcomes.

### Information transformation, feedback, reciprocity over time

Though the model represents reported constructs to facilitate healing relationships, there are foundational values that represent these relationships and the resultant healing pathway. We review these values in order to encapsulate and contextualize the data elements. Information transformation, feedback, and reciprocity are clearly underlying principles which warrant consideration in the context of illustrating the Healing Pathways Model [56]. These constructs operationalize mechanisms for contextualizing the patient-provider relationship through interactions, feedback, the ‘give and take,’ and outcomes that occur and change over time between patients and providers. Patients and providers communicate to produce and receive input, which is processed into an output which generates feedback. Said feedback is again received as input – creating an ongoing cyclical reciprocal pathway [57]. The recursive nature of feedback results in information transformation and can modify the output of the system to promote system proficiency (e.g., healing). However, when the flow of information is disrupted, as when patients and providers engage in ineffective communication or non-reciprocal interactions, patients experience imbalance and the opportunity for mutual participation/partnership building is lost. It is imperative that patients have time to disclose their concerns, ask questions, and engage in meaningful dialogue. Over time in the patient-provider relationship, and within a single face-to-face visit, providers can manage time to meet patient needs by effectively navigating the Healing Pathway.

### Patient & provider independent factors

Data findings informed the domains of the provider approach for facilitating healing; these factors describe how providers perceived their role as facilitators and partners to effectively engage the whole person in creating a healing pathway. Their self-awareness of creating a compassionate presence, creating a healing space, as well as cleansing and committing to self-care, were all a collective approach to support their own wellness, and help them maintain their capacity to facilitate healing.

Additionally, data clearly indicate sensitivity to cultural context, and as such, The Healing Pathway Model, as a culturally sensitive model, accounts for the cultural context of health encounters. The model suggests that patient and provider factors such as race/ethnicity/culture, age, sexuality, socio-economic status, education, language, gender, and personal history can influence the medical encounter and subsequent outcomes. Within the context of this model, patients and providers engage in health interactions with predetermined beliefs and attitudes that influence the pathway and outcomes of the patient-provider relationship. Open communication about beliefs and attitudes can help patients and providers not only promote individualized care but also assist in co-creating treatment goals and outcomes.

### Outcomes

The final component of the Healing Pathway Model was identified as *Outcomes*, which were identified through observations in the existing literature of healing and issues that prevent healing. *Healing* in this context does not equal cure, but patients may experience *symptom relief*. Healing relates to *whole person outcomes*. Whole person outcomes address wellness and potential rather than finding a cure [47]. To provide contextualization within healthcare systems, factors such as *patient and provider satisfaction*, *compliance/adherence*, and *resource use* are appropriately reflected in the Healing Pathways Model.

## Discussion

The proposed Whole Person Health oriented constructs and Healing Pathway Model illustrate the interdependent dynamics of the patient-provider relationship in the co-creation of healing, as perceived by providers. The conceptually driven pathway model provides a contextualized organization of relevant constructs and assumptions of the patient-provider relationship within the context of Whole Person Health systems. Previously published models and instruments have addressed aspects, including satisfaction, trust [58], well-being and wholeness [59], understanding [60], confidence [60], and global satisfaction [58]. There is currently not a single comprehensive model that guides development of tools and trainings that address critical aspects of continuous healing relationships. To date, patient-provider relationship building efforts have been relatively limited to conventional/biomedical settings and have lacked standardization.

This Whole Person Health oriented Healing Pathway Model echoes key constructs from previously published models and frameworks, including the Rede Model (e.g., engagement and rapport) and work from Watson (e.g., love/compassion) [9, 40, 43], and acknowledges foundational published work; however, this work distinguishes itself in two important ways: first, it is qualitatively driven by interdisciplinary elements that are relevant to the co-creation of a healing relationship; and second, this model reflects the emergence of soft skills for creating an authentic human connection, which is important in many contexts, but particularly in the case of patients with a history of traumatic experiences, such as veterans [61, 62].

The Whole Person Health oriented Healing Pathway Model provides a dynamic illustration of the complex, yet human, aspects of engaging in the patient-provider relationship, which has been repeatedly linked to a diverse range of interpersonal and health outcomes [63]. More importantly the model should remind us that as each component is influenced, whether by the patient, provider, environment, etc., similarly to a multi-level public health model [3], the pathway is significantly affected, likely affecting other components. This ever-evolving relationship can produce a myriad of outcomes over time through information transformation and feedback. Therefore, providers may benefit from being aware of, and optimizing, components of the patient-provider relationship to promote the principles of Holistic Medicine, which are intended to optimize the healing pathway for the whole person.

This descriptive study, from a whole person perspective, informed our understanding of the interpersonal aspects of healing across a diverse group of health professionals from different regions of the country. This work moves beyond previous models in that it provides specific soft skills for health professionals to develop in order to facilitate healing. Furthermore, the model places value on emotional and energetic constructs within the context of care. These constructs have been minimized in previous models. Moving forward, the illustrated constructs of healing could be used by providers across disciplines to facilitate the whole person healing pathway with their patients.

### Limitations and implications

The descriptive qualitative methods used in this study provided a rich dataset that resulted in a comprehensive perspective of conventional and CIH providers’ experiences in facilitating healing; however, limitations should be noted. First, findings may reflect bias due to the nature of participant self-selection to participate and the use of snowball sampling. However, this sample – which, collectively, provided saturation in results – was purposively recruited to represent the perspective of both conventional and CIH providers. Second, the development of the Healing Pathway Model, though informed by a dynamic theoretical perspective, was developed based on a single dataset, which may present limitations based on the self-reports of the participants. Third, reflexivity, or the lack thereof, is a major factor in qualitative research and can protect against researcher bias. As such, data and inferences were examined by multiple team members to minimize researcher bias. Fourth, only provider perspectives about the healing pathway were obtained in this study. Future studies should include the voices of both providers and patients.

#### Implications

This research describes the healing pathway as described by a diverse cohort of CIH and conventional providers. Findings suggest that quality-of-care delivery depends on interpersonal factors and behaviors, not on the type of provider. This philosophical approach holds merit in biomedical healthcare models but is particularly valuable in the context of delivering a whole health model of care while caring for the whole person. Providers may benefit from recognizing themselves as a therapeutic agent in interactions with patients. By intentionally capitalizing on interpersonal dynamics, providers have an opportunity to develop continuous healing relationships and improve patient outcomes.

The implications of this study also extend to professional education and training. Aspects of facilitating the healing pathway should be identified and cultivated throughout the professional development and care delivery process. Although foundations of the healing pathway are well established in health-related professional development (e.g., resource management), soft skills, like appropriate expression of love and compassion, are not only neglected, but in many cases discouraged. Progressive approaches to health and healing have come to recognize the therapeutic components of expressions of love and compassion in the healing pathway [64, 65].

Future work should complement the provider perspective by validating concepts from the patient perspective. Additionally, future studies should examine the performance of the Healing Pathway Model in different contexts, inform the development of a patient-provider assessment, and examine the model’s potential to impact patient and provider outcomes.

## Conclusion

As the culture of medicine leans into a consumerist model, interpersonal dynamics will continue to rise to the surface as a critical aspect of delivering high quality care to support the facilitation of healing, and dying. Provider perspective data across disciplines informed the development of a conceptually driven healing pathway model to inform how to leverage patient-provider relationships to facilitate healing. As efforts continue to advance the science of interpersonal aspects of healing, these data are relevant to understanding the factors of continuous healing relationships between patients and providers. These efforts can inform the identification and standardization of factors relevant to whole health care systems implementation, processes, and outcomes – from the perspective of the patient-provider relationship. Researchers can utilize this model as a framework to identify points of inquiry to better understand the complex pathway of the patient-provider relationship, and health related outcomes. In the current climate of healthcare systems, with shifting sands of when, how, and who patients receive care from, these data warrant examination of how the patient/provider interpersonal relationship facilitates healing beyond traditional clinical care practice.

## Supplementary Information

Supplementary Material 1.

## Data Availability

Data can be made available upon reasonable request.

## Abbreviations

CIH: Complementary and integrative health
IRB: Institutional review board
