# Supplementary material for: Enhancing patient-provider relationships with a whole person oriented healing pathway model
Source: BMC Health Serv Res. 2025 May 13;25:682. doi: 10.1186/s12913-025-12858-8 (PMC12070668; doi:10.1186/s12913-025-12858-8)
Supplement: Supplementary file 1 — Supplementary Material 1. [file 12913_2025_12858_MOESM1_ESM.docx]

**Appendix A. Interview protocol.**

| **Item** | **Probes** |
| --- | --- |
| How would you describe your practice and patient population? | How would you describe a typical patient visit? |
|  | How long is a typical visit? |
|  | What usually occurs during a visit? |
|  | Is there a difference between new patients and someone you’ve seen before? If yes, How are they different? |
| What is your experience as a practitioner? | How does it feel to work with your clients? |
| Can you describe any preparation you go through when preparing to work with a patient? | Can you describe any internal preparation you might experience when preparing to work with patients? |
|  | Can you describe any bodily feelings you might experience when preparing to work with patients? |
|  | Can you describe your thoughts when preparing to work with patients? |
| How do you get a sense of your patients’ needs? | How do you determine what your patients’ priorities are? |
|  | How do you determine the *individual* needs of your patients? |
| To what extent do you follow specific protocols/treatment plans, as opposed to your intuition? | What percentage is intuition vs. protocols and treatment plans? |
|  | What do you think about in reference to *reading patients* (their body)? |
|  | Do you receive visions or guidance when consulting/treating your patients? If yes, does this information inform your practice? |
|  | Do you receive visions or guidance before (or after) consulting/treating your patients? If yes, does this information inform your practice? |
| Can you describe any *shifts* you’ve seen patients experience when receiving care from you? | Is there a difference between acute shifts versus shifts which might occur over time? If yes, can you please describe this difference you have experienced with patients? |
|  | How do you know when patients have experienced a turning point in their healthcare process? |
|  | Do you find there are differences in language before and after turning points in their healthcare process? If yes, can you talk to me about these differences in language? |
| Thinking of your best experiences with your patients, can you describe to me what that process of healing was like for you as the practitioner? | How do you view your role in the healing process? |
|  | How did you co-create a healing experience with your patient? |
|  | Do you experience any feelings or emotions towards your patients? |
| In summary, what do you do to facilitate healing with your patients? | How do you know when what you are doing is creating healing? |
|  | How do you help patients get to turning points in their healthcare process? |
|  | On the other hand, what do you find gets in the way when facilitating healing with patients? |
|  | How do you understand and respond to patients’ emotional reactions? |
|  | How do you know your patients understand what you tell them, the information you give them, what you’re doing, and why you’re doing what you do when treating them? |
| Do you see any similarities between your personal life and what you’re dealing with, with patients? | Is there a connection between your personal issues and issues arising in your practice?) |
| Do you do anything to cleanse yourself after working with patients? If yes, can you tell me about your cleansing routine? | N/A |
| After talking about activating pathways to healing and co-creating healing with patients, do you feel your training covered the topics we’ve discussed today? | If no, do you feel additional training would be appropriate? If yes, what should additional training address? |
|  | What would training, pertaining to activating healing pathways with patients look like? |
